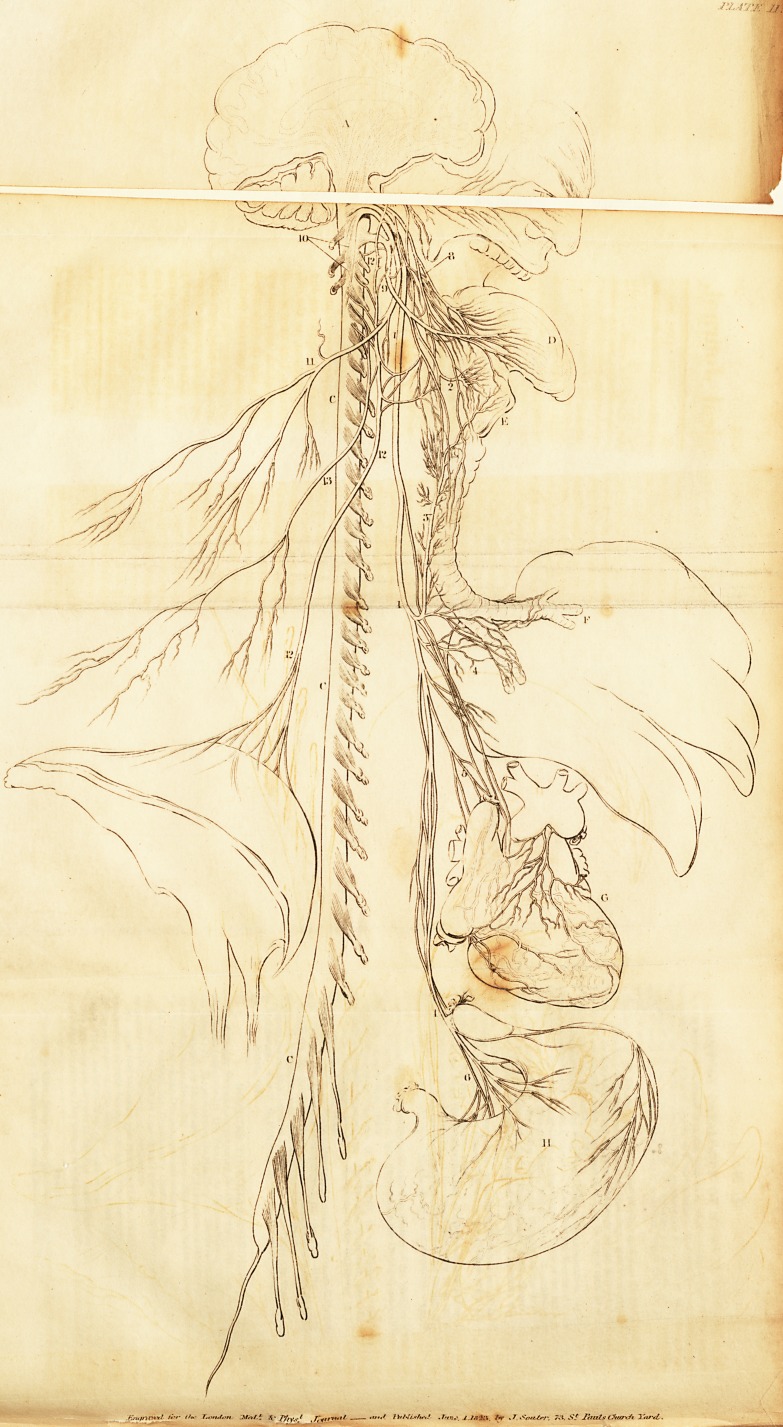# Second Part of a Paper on the Nervous System

**Published:** 1823-06

**Authors:** John Shaw


THE LONDON
### Medical and Physical Journal.

6 OF VOL. XLIX.] JUNE, 1823. [No. 292.

For many fortunate discoveries in medicine, and for the detection of numerous errors, the world is indebted to the rapid circulation of Monthly Journals; and there never existed any work, to which the Faculty, in Europe and America, were under deeper obligations, than to the Medical and Physical Journal of London, now forming a long, but an invaluable, series.—RUSH.

### ORIGINAL COMMUNICATIONS,
### SELECT OBSERVATIONS, &c.

ART. I.—*Second Part of a Paper on the Nervous System.* By JOHN SHAW, Esq.

IN compliance with your request, and that of other friends, I shall now endeavour to fulfil my promise of giving a slight sketch of the *Superadded Nerves.* But, as a considerable time has elapsed since the publication of my last communication to your Journal, I shall, before entering upon the subject again, make a short recapitulation of the characteristic distinctions of the *Regular*, *or Original*, *Nerves.*

Under this class were comprised the fifth, or trigeminus, the suboccipital, the seven cervical, the twelve dorsal, the five lumbar, and the five sacral; in all, thirty-one pairs of nerves.*

To all of these nerves the following characters are common: —an origin by two distinct sets of filaments; a ganglion on one of the roots; a distribution, in a lateral direction, to the different parts of the body. We find, moreover, that they are all compound nerves; being the media through which the voluntary motions common to all animals are ordered, and through which the sense of touch and common sensibility are bestowed on the different parts.

When the trunk of one of these nerves is divided, not only are the muscles to which it goes, deprived of the power of executing certain motions, but the sensibility of the part is entirely destroyed; but, if only the set of filaments by which the nerve arises from the anterior column of the spinal marrow be cut, the voluntary power over the muscles alone is lost: whereas, if the posterior set of filaments be divided, the sensibility of the part is destroyed, while the voluntary power continues unimpaired.†

* See the Plate in the Number of this Journal for December 1822, and C, C, C, in the accompanying plan.

† It is now quite unnecessary to state any circumstances to prove, that the sole merit of the discovery of the distinctions between the characters and functions of

It may be also stated, that the facts furnished by the examination of the structure of the spinal marrow, and the results of experiments, have induced Mr. Bell to believe that the different fibres or striæ of nervous matter, minister to the same order of functions through all their length. For example,—if we trace the nervous cord of that part of the crus cerebri which gives origin to the third nerve, or motor oculi, down to the cauda equina, we shall find that, through its whole course, it is connected with nerves of the same function or character. We may trace from it the third, the sixth, the ninth, and the anterior roots of the spinal nerves; and nearly all those nerves can be *proved* to regulate the voluntary powers.

After having arranged the nerves just described under one class, we shall find the intricacy of the nervous system much diminished; and, in attempting to unravel it farther, we shall arrive at the following important conclusion,—that all the other nerves (with the exception of the sympathetic,) have relation to parts which may be considered as superadded to the original frame of the body; and that their branches and connections are numerous or complicated, in proportion to the variety of functions which the superadded organs perform.

I shall not now enter upon the description of the nerves of sense, or of those which are distributed to the parts within the orbit,* although all of them may be considered as superadded nerves, but confine my observations to those exhibited in the accompanying plan; which, it will be acknowledged, are the nerves that, by their distribution and connexions, mainly contribute to the intricacy of the system.

These nerves will be found to be chiefly intended for the purpose of regulating the functions of such parts as are more or less connected with respiration. They are the *portio dura* of the Seventh; the three divisions of the Eighth,—viz. the *glosso pharyngeal*, the *par vagum*, and the *spinal accessory;* the Ninth, or *lingualis;* the *phrenic;* and the *external respiratory*.

I shall give a short description of each of these nerves; and, by references to comparative anatomy, endeavour to show upon what circumstances their existence and their complication depend.

First, of the PORTIO DURA of the SEVENTH, or respiratory nerve of the face, marked in the Plan 7, and seen passing forward from the ear upon the muscles of the eye, nose, and mouth.

---

the roots of the spinal nerves, is due to Mr. Bell; for it is not only now publicly acknowledged in this country, but has also been fully admitted by M. Majendie, to whom the merit had erroneously been given by some gentlemen.—See my communication in the Number for October.

* The nerves of the orbit are very complicated; but their intricacy has been, in a great measure, unravelled by the description given of them in two papers which have this season been presented to the Royal Society by Mr. Bell.

Although this be very generally called the Facial Nerve, it is not found upon the face unless there be some consent of motions established between the muscles of the face and the respiratory organs. Thus, in fishes, the nerve, instead of being distributed on the face, passes to the muscles of the gills; and, in the game-cock, we find it passing principally to the muscles below the jaw, and to those which raise the feathers of the neck.

Although the nerve be generally described as rising along with the auditory nerve, yet we find in many animals that it rises with the branches of the eighth: this is particularly observable in fishes and in birds.*

The proportion of the portio dura, or facial respiratory nerve, to the fifth pair, is greater in man than in any other animal. In the monkey, its proportion to the fifth is diminished; but still in this animal it is more complicated than in the dog, or any of the carnivorous animals, the intricacy of the branches being apparently in proportion to the number of the muscles of expression. From the lion, the dog, and the cat, we may descend to the horse, ass, and cow: in the latter animals, there is a marked difference in the distribution of the nerve, from that of either the monkey or the dog; for, excepting a few branches, which pass to the muscles of the external ear and to the eyelid, the whole of the respiratory nerve is confined to the muscles of the nostrils and side of the mouth.

There are, however, some varieties in the classes of the graminivorous animals. In the gazelle, sheep, and deer, the distribution of the nerve is still more simple than in the horse; while in the camel it is more profuse, and is, in this respect, intermediate between that of the carnivorous and the graminivorous animals. Indeed, the expression of the enraged camel is sufficiently ferocious; and the manner in which he shows his tusks, when dying, is very similar to that of a carnivorous creature. Although we are told by those who have seen an elephant in a rage, that he is most sublime and terrific, still the anatomy of the portio dura of this animal leads me to suspect that the expression of rage, however terrible, must be quite different from that of the ferocious snarl of the lion. In the face, it must be in a great measure confined to the contortions of the proboscis, and to the eye; for, except a few branches to the eyelids, the distribution of this nerve of

* In the *Leçons d'Anatomie Comparée*, a description is given of the origin and course of this nerve in the calf. The connexions between it and the par vagum under the ear, though the same, as in other mammaliæ, are there described as the origins of the *facial* nerve. Since the names *portio dura* and *facial nerve* are used indiscriminately in this country, it is probably in consequence of this description that some gentlemen have described the portio dura in the calf as rising with the par vagum.

respiration and expression is confined almost entirely to the proboscis.

As the anatomy of the nerves of the trunk of the elephant has been hitherto very incorrectly given, I shall here offer a description of them, from notes taken at the dissection of a young one.

The portio dura was found emerging from the parotid gland, as in other mammalia. It gave off some descending branches to the neck, but passed from behind the jaw to the proboscis, almost as an entire nerve, and of the size of the sciatic nerve in man. In its course, it had only given some small branches to the muscles of the eye, to those of the ear, and to a small muscle which corresponds with the platysma. Before it passed into the substance of the proboscis, it united with the second division of the fifth pair, which comes forward from the infra orbital hole, in two large branches. The two nerves, being then closely united, passed between the layers of the muscles which form the greater mass of the trunk. The portio dura became quickly diminished in size, as it gave off its branches in great profusion to the muscles; but the fifth was continued down, as a very large nerve, to nearly the extremity of the trunk, in this respect resembling the nerves to the fingers in man. On making sections of the proboscis, near its extremity, a great number of the branches of the fifth were seen in its substance.

A few branches of the portio dura ran to the valvular apparatus in the upper part of the trunk; but this peculiar structure was supplied principally by a branch from the fifth pair, which winded round under the orbit.

If we compare the anatomy of the facial respiratory nerve in the various classes of birds, we shall find its distribution to be analogous to that of the same nerve in the different tribes of quadrupeds. In the game-cock, a few branches of the nerve pass to the muscle connected with the loose skin under the jaw, which is dilated in crowing; the greater number being distributed on the muscles of the neck, which cause the elevation of the feathers when this bird puts himself in an attitude for fighting. But in the duck, which, when enraged, has little or no power of expression, the same nerve is not larger than a cambric thread, and passes only to the muscle under the jaw.

As so many observations are already before the public descriptive of the effects produced by cutting this nerve,* I shall only submit the question, whether the numerous experiments that have been made do not, when taken into account with the

* See the Philosophical Transactions, and the communication by me to this Journal in December last. The question of the apparent sensibility of this nerve is alluded to in the Number for October.

comparative anatomy of the nerves of the face, prove that the use of the portio dura in man, is to combine and regulate the action of all those muscles of the face which are *in any way* connected with respiration.

The nerve next in order is the EIGHTH. We should consider the three nerves of which it is composed as distinct, although they be united in function.

The first is the GLOSSO PHARYNGEAL, and is marked 8 in the Plan. The origin and distribution of this nerve distinctly point out its use,—viz. that of combining the actions of the tongue and pharynx in deglutition. Its power over the pharynx has been shown by several experiments, the results of which were very curious, and corroborative of the views deduced from comparative anatomy.

The next, the PAR VAGUM, is perhaps the most interesting nerve in the body. It is marked 1 in the Plan. Two distinct branches pass from it to the larynx;—viz. the SUPERIOR LARYNGEAL, marked 2; and the INFERIOR LARYNGEAL, or RECURRENT, 3. We see also the PULMONIC PLEXUS, 4; the CARDIAC PLEXUS, 5; and the GASTRIC PLEXUS, or corda ventriculi, 6. The ŒSOPHAGEAL PLEXUS, though not marked by any figure, may be easily discovered. When we examine this nerve minutely, we find that it is not only intimately connected with all the nerves seen in the accompanying Plan, but also with the Sympathetic.

To go into the full consideration of the anatomy and connexions of the par vagum, would far exceed my limits; but here I may observe, that, unless we examine the comparative anatomy of this nerve, we shall be very apt to draw erroneous conclusions from experiments made upon it, in the mammalia. Indeed, if we examine what has been deduced from many of the experiments lately made upon the par vagum, we shall be led to suspect that the experimenters did not take into consideration the fact, that this nerve exists only where there is a necessity for a combination between the functions of the stomach and of the lungs. For example, it has been attempted, by experiments, to prove that the secretion of the gastric juice depends on the par vagum; forgetting, apparently, the well-known fact that, in many animals which have the power of digesting very crude substances, there is no nerve of this kind.

While upon this subject, I may take the opportunity of stating my belief that certain opinions, which are at present held upon the functions of the viscera of the thorax and abdomen, are the means of keeping up many erroneous notions on the uses of the nerves. To me it appears that the principal object of many of the late experiments has been to discover the power which enables the heart to contract, or the glands to secrete. This power has, I believe, been generally ascribed to the nerves which are seen passing into these viscera; but, when we find that a *stomach* which secretes gastric juice, a *pancreas* saliva, a *liver* bile, a *kidney* urine, are all furnished with the same nerves, and that these are not only the nerves of the lungs and of the heart, but also of the muscles of the pharynx and larynx, we are, I think, forced to conclude that this nerve is not for conveying a power or principle to these parts, which shall enable them to perform their several offices. Indeed, we may even believe that these organs have a power independent of the par vagum, or perhaps of the brain, since we find them capable of performing their several functions, not only in animals so low in the scale of existence as to have neither brain nor nerves, but even in monsters where a great part of the nervous system is deficient.

The attempt to discover what is the principle by which the different glands are empowered to secrete certain fluids, would be fruitless: but still this should not prevent us from investigating the laws upon which the due performance of those actions depend.

I am aware that this is a very difficult subject; but, as certain clear and demonstrable facts can be substantiated, I may be permitted to allude to the question, though in a cursory manner.

It will probably be acknowledged, that the body of the more perfect animals is so constituted that each organ has a power, to a certain extent, of performing its peculiar function; but that this function will not be properly performed, unless the combination or relation of the organ to the other parts of the body be perfect and uninterrupted; and, moreover, that the undue performance of the functions of one organ will have a certain effect upon those of others. Still it does not follow that any virtue is actually conveyed from one viscus to another, but only that they are united together so as to constitute a circle of actions mutually dependent on each other. Indeed, we know that the secretion of gastric juice by the stomach is imperfect, unless the actions of the lungs be properly performed; and, on the other hand, that our endeavours to resuscitate an animal apparently drowned, will fail if there be poison in the stomach.

There can be little doubt that the combination between the different organs is kept up by nerves. In the mammalia, we see the par vagum pass from the lungs to the stomach; and we may assume that it is the bond of union between their functions, since neither those of the one viscus nor of the other are perfectly performed if the nerve be divided.

Seeing that the gastric juice is not secreted when the par vagum is cut, we are at first view inclined to infer that the power of secretion depends on the par vagum; but, when we find, by the investigations of comparative anatomy, that the stomach may be entirely independent of such a nerve, we are compelled to give up this opinion. Indeed, the arguments in favour of it are still further weakened by finding that if, after the par vagum has been cut high in the neck so as to disturb the functions of the lungs, respiration be assisted by any artificial means, the functions of the stomach will be partially restored.

Perhaps the only legitimate conclusions we can draw from the observation of such facts, are that the par vagum is the medium through which the several important organs are knit together and associated in their function, and by the injury of which the organs themselves are hurt and deranged. Digestion, respiration, and circulation, are not separate functions, but the different stages of one great operation, "nutrition," necessary to life; and for this purpose the organs are bound by sympathies, which cause them to act in unison, and by which they become mutually dependent. Hence the injury of one has an effect upon the others, and the destruction of the medium of connexion disturbs the whole economy.

In this inquiry it is quite admissible to examine how far those who have held different opinions have been correct in their views of the anatomy of the nerves, and particularly in the assumption of the data from which they have drawn their conclusions regarding the uses of the par vagum. In doing so, we shall discover that the same deductions have been drawn from experiments where the par vagum and sympathetic have been cut, as from those where only the par vagum was divided.*

I shall not make any further observations at present upon this question, but submit, that since, in the more complicated animals, (as in the mammalia,) the par vagum passes to the throat, the larynx, the heart, the lungs, and the stomach, its use is probably to connect and combine, into one great system, these several organs,—each of which has the power of performing, to a certain extent, its own peculiar function. And hence it naturally follows, if the nerve be divided, the connexion between all the organs, and also betwixt them and the external muscular apparatus, upon which the perfection of the economy of each depends, will be destroyed.

Although I will not at present enter into the full consideration of the SYMPATHETIC, I shall make a few observations on the prevailing opinions regarding its anatomy and uses.†

---

* The par vagum and sympathetic are so closely united together in the neck of the horse, that it is exceedingly difficult to separate them, even in the dead animal.

† On another occasion, I propose to show that the anatomy of the sympathetic has been so erroneously described, not only in the thorax, but also in the neck and head of many of the lower classes of animals, as to entitle us to deny that any of the modern theories on the uses of this nerve are founded on correct views of its anatomy.

It will at once be admitted, that the descriptions most commonly given of late of this nerve are copied from the works of Bichat. Now I do not hesitate to state that the description, as given by Bichat, is incorrect; and that it does not correspond with what is found upon dissection, nor with that given by the most eminent anatomists who preceded him.

If we examine the manner in which the nerves arise from the spinal marrow, we shall find that each nerve has not only a double root,—i. e. one from the anterior, and the other from the posterior column of the spinal marrow,—but that they are also all united with, or give off a branch to the sympathetic. This union, or origin, of the sympathetic from the spinal nerves appears to have been entirely overlooked by Bichat. We may however presume that, had he lived, he would have given up the idea of considering the sympathetic as a part entirely distinct from the system of the spinal nerves; for it is a striking and curious fact, that, in the edition of his "Anatomie Descriptife," published in 1802, the editor says, "Nous reprenions ensemble le systême nerveux des ganglions et c'étoit le soir même ou nous avions commencé le ganglion cervical superieur que Bichat fit cette funeste chute qui determina sa dernière maladie."

It is, perhaps, not assuming too much to say, that since Bichat was incorrect in his views of the anatomy of the sympathetic nerve, it follows that not only his own ideas on the ganglionic system are untenable, but that also all the conclusions from experiments, which have been instituted in the belief that his observations were correct, are also liable to objections. I shall not here state the conclusive arguments that may be offered in contradiction of the opinion, that the system of the sympathetic is similar to the nervous cords seen in the lower animals; but I shall merely ask how far the theory, that the actions of the heart depend more upon the sympathetic than on any other nerve, can be correctly founded, when it is easy to demonstrate that, at every intercostal space, the heart is united, through the sympathetic, with the spinal marrow. The observation that the sympathetic has been found perfect in monsters in which the spinal marrow was deficient, affords no argument in favour of the sympathetic being isolated from the other parts of the nervous system; for, in such creatures, we generally find also the spinal nerves, which are by all acknowledged to have their origin from the spinal marrow.

The difficulty of discovering the power by which the various glands secrete the different fluids, has been already noted. The same difficulties are presented when we inquire into the causes of the actions of other important organs. For example, if we cut out the heart, and thus at once, in the most effectual way, separate it from the nervous system of the body, we shall find that it will contract and dilate in its own peculiar manner; and if at the same time we remove part of the stomach or intestines, we shall see their muscular fibres act in quite a different manner from those of the heart, although both parts are supplied with the same nerves. More examples might be offered, but these are sufficient to show that each part is endowed with a power, the discovery of the nature of which is probably beyond our reach. If it does reside in nerves, we may, from the facts just mentioned, conclude that it is not derived from the brain, or from the nerves which we see passing to the several parts, but that it exists in a nervous substance which cannot by any art be separated from the organ. Let us therefore, in imitation of those who have made the greatest discoveries in other sciences, confine our inquiries to the laws by which the functions of the several organs are governed.

Before leaving the subject, I ought to state that the greater number of the above observations on the functions of the par vagum and sympathetic, were published two years ago, in the first edition of my "Manual of Anatomy."

The last division of the eighth pair is the SPINAL ACCESSORY; it is marked 11 in the Plan. As this is a very curious nerve, I shall go at some length into the description of it.

The spinal accessory arises from the cervical portion of the spinal marrow; but, instead of collecting its branches to go out by the side of the vertebræ, like the cervical nerves, it shoots upwards in the theca of the spinal marrow, enters the skull, and joins the glosso pharyngeal and par vagum; from which, it has its term of accessory to the eighth pair. We see the roots of this nerve as far down as the fourth cervical nerve. These roots arise neither from the posterior nor from the anterior column of the spinal marrow, but betwixt the posterior roots of the cervical nerves and the ligamentum denticulatum. The origins (marked 10 in the Plan,) come off in one line, in the direction of the roots of the eighth pair, and of that nerve which has been proved to be the respiratory nerve of the face, —viz. the portio dura. In its ascent, the accessory nerve is attached to the posterior root of the first cervical nerve.

The nerve, having ascended through the foramen magnum, passes out of the skull, associated with the nerves constituting the eighth pair, and in the same sheath with them: they all go out through the foramen lacerum, and by the side of the jugular vein. In this course the accessory nerve is divided into two. One of these divisions joins filaments of the par vagum, and these again send branches to the glosso-pharyngeal nerve; and sometimes a twig may be seen going to the lingualis medius. The next exterior division of the accessory nerve descends behind the jugular vein, and comes forward and perforates the mastoid muscle. In its passage through the muscle, it sends off branches which course through its substance; and if (as sometimes happens, though rarely,) the nerve does not pass through the muscle, these branches are, notwithstanding, invariably given to it.

When the nerve has escaped from the back part of the mastoid muscle, it forms a communication with that branch of the third cervical nerve which ascends behind the muscle, and nearly at the same time it is joined by a branch from the second cervical nerve. The superior respiratory nerve now descends upon the neck, and begins to disperse its branches in regular order to the trapezius muscle: four or five branches take their course to that muscle, separate into minute divisions, and are lost in its substance. One more considerable division, being the lowest of these, is joined by a long descending branch of the second cervical nerve. Increased by this addition, it descends under the trapezius and behind the clavicle. Following this descending branch, we shall find it exclusively attached to the trapezius. Behind the scapula it is again joined by branches from the spinal nerves; and here a sort of imperfect plexus is formed, from which divisions of the nerve, still descending, follow the lower edge of the muscle, and are finally dispersed among its fibres.

We may recapitulate, by saying that this nerve arises from the same column as the respiratory nerves; it takes a most intricate and circuitous passage to form a junction with nerves which we know to be of that class; it sends branches to join the nerves of the tongue and pharynx; it sends branches to the larynx, in company with those of the par vagum; it then crosses the great nerves of the neck, passes under the spinal nerves, and is then distributed principally to the mastoid and trapezius muscles.

By observing the facts furnished by comparative anatomy, and the results of experiments, we shall probably form a correct notion of the uses of this very curious nerve, which does not seem to have as yet attracted the attention of anatomists so much as it deserves.

If an animal does not perform part of the act of respiration by muscles which run from the skull to the chest, no spinal accessory is found. The truth of this observation may be shown by the dissection of any of the larger birds; but the most extraordinary proof is to be found in the neck of the camel. The construction of the neck of this animal is like that of birds; there being a succession of short muscles along the side of the neck and attached to the vertebræ, but no long muscle passing from the jaw to the sternum, to assist in breathing, as in other quadrupeds. It is most probably in consequence of this variety in the muscular apparatus, that the arrangement of the nervous system of the neck is in this animal similar to that in birds.

In the dissection of a courier camel, or *maherry*, which was brought from the interior of Africa by Captain Lyon, as a present to his Majesty, we found that the nerves of the neck, in their number and distribution, resembled those of the swan, much more than of a horse or bullock, and particularly in the spinal accessory being either deficient altogether, as in birds, or quite different to what is found in the greater number of quadrupeds.

While last in Paris, I was told that there was a preparation of the brain of a camel in M. Cuvier's collection, in which the spinal accessory nerve might be seen; but, on examining the preparation, I could find no trace of the nerve. There were some little filaments pointed out to me as the origin of the nerve, but to their being so, I could not assent. If these fibres (which, however, in the state the preparation then was, were very obscure,) be compared with the origin of the spinal accessory, even in the sheep, we shall be forced to conclude that, if there be a nerve of this kind in the camel, it is so small as to form an additional proof that the character of the nerve depends on a particular conformation of the muscles of the neck.

I must refer to the papers by Mr. Bell in the Philosophical Transactions, for many of the arguments proving the particular influence which this nerve has over those muscles to which it is distributed: here I shall only state, that, by cutting the spinal accessory, they are paralysed as muscles of respiration, though they still retain, through the media of other nerves, their powers of raising the head, &c.

The first experiment made upon this nerve was very conclusive. The par vagum of an ass was first divided, with the intention of causing difficult and laboured respiration. When all the respiratory apparatus was in great agitation, and when the sterno maxillaris (the same as the sterno cleido mastoideus in man,) was especially in action, the spinal accessory nerve was divided. In an instant the sterno maxillaris ceased to act as a muscle of respiration; but, when the animal struggled to get free, it became rigid, showing that, through the plentiful supply of spinal nerves, it still retained its office of moving the head and trunk.

The next nerve, LINGUALIS, or NINTH, (marked 9 in the Plan,) is distinctly a *superadded nerve*, passing to the muscles of the tongue; the number and intricacy of its branches being dependent on the complication of the functions of the tongue.

I cannot offer a better example of this, than the comparison of the nerve in the dog with that in the horse or ass: in the dog, which breathes through the mouth, and uses the tongue very much in feeding, the branches are very numerous, particularly those which correspond to the descendentes in man; while in the ass, which does not breathe through the mouth, nor use the tongue in the same manner as the dog, the distribution of the branches is very simple, and the descendens is so small as to be with difficulty discovered.

The motions of the tongue are principally, though not altogether controlled by this nerve. I cut both the ninth nerves in a dog. After the operation, putting his mouth into the dish, he appeared to lap the milk that was offered to him, but the power over the tongue was so far destroyed that he could not throw any of the milk down his throat. When meat was put fairly to the back part of his mouth, he swallowed it. Although the power over the tongue in feeding was entirely destroyed by cutting these nerves, he could still move it in several directions, and there was no change produced in his barking.

The only nerves that remain to be arranged are the Phrenic and that which has been called by Mr. Bell the External Respiratory.

It is unnecessary to say much on the functions of the PHRENIC, (marked 12 in the Plan,) for, in all ages, it has been considered as the nerve which regulated the actions of the diaphragm, or great internal muscle of respiration. I shall only remark, that, although its origin is generally described as from the third and fourth cervical nerves, it will be found to unite with the portio dura, par vagum, and ninth, or lingualis; and thus we have, in its connexions, as in the results of experiments, proofs that it is one of the great bonds of connexion between the different classes of the respiratory muscles.

The next nerve, EXTERNAL RESPIRATORY, (marked 13,) until described by Mr. Bell, was scarcely attended to by anatomists. I can recollect, long before Mr. Bell made the discovery that the portio dura and spinal accessory were nerves assisting in certain acts of respiration, he used to insist upon this nerve being always demonstrated with the phrenic. Indeed, when we examine its origin and course, we are astonished that it should have been so long neglected. It comes out from the fourth and fifth cervical nerves, and often it is connected with the phrenic. It diverges somewhat from that nerve; because, instead of descending within the chest, it falls over the ribs, and descends in a distinct flat trunk upon the outside of the chest, to be distributed to the serratus magnus anticus. This muscle has also nerves from the spinal marrow, because it has to unite in the motions of the frame in locomotion. But the long descending nerve is a respiratory nerve; which is evident from its origin, course, and destination: in its origin and course it is like the diaphragmatic nerve, and in its destination also, since it is given to a muscle necessary to full respiration.

I hope that the slight sketch offered here, will be acceptable to one class of the readers of the Medical Journal; for I have had ample opportunities of observing the effect which the late discoveries have already made on the minds of our students. Instead of now considering the nervous system as a labyrinth, into the mazes of which they dare not venture, they enter upon its investigation with the conviction that it will prove more satisfactory and more interesting than that of any other department of the science of anatomy. By examining the nervous system in animals less complicated in their structure than man, they are at once enabled to comprehend the general arrangements; and, from the nomenclature of the principal nerves corresponding with their functions, they can, in a short time, understand that which, to students of former years, was matter of the utmost labour.

They who are anxious to investigate the anatomy and physiology of the respiratory nerves, will find the inquiry much facilitated by the dissection of some of the lower animals. But it is necessary that they should examine the parts themselves, and not be satisfied with the descriptions which are at present generally given: if they do so, they will find much cause for objecting to the descriptions of the nervous system which are copied from the works of the French anatomists.

I have already pointed out two curious facts which have been omitted by these gentlemen in describing the nervous system of the two largest quadrupeds, the elephant and camel; but it would be ridiculous for me to ground my objections on the facts to be observed in those animals, since very few can have an opportunity of observing them. I shall therefore beg my readers to examine the nerves in the calf or in birds, or in the lobster or the snail, and compare what he finds with the descriptions generally given, before he comments on the justice of my criticism. If the criticism be justly founded, the importance and necessity of it must be obvious.

In conclusion, I may state, as a general observation, that in the greater number of the mammalia, the nervous system within the thorax, is nearly the same as in man. In the abdomen, its structure is complex, in proportion to the intricacy of the intestinal canal: hence it is less simple in the ruminating than in the carnivorous animals or in man. In the neck and face, it bears the same relation to the parts. If an animal has little or no power over the voice, and breathes only by the nostril, as the horse or ass, the arrangement of the respiratory nerves is particularly simple; and, if we compare the nerves on the neck of the ass with those of the dog, which breathes principally by the mouth, we shall be enabled to understand the apparently complicated distribution of the nerves to the human neck. When this structure is thoroughly understood, it will be clearly seen how well these nerves are adapted to admit of equal facility of breathing by the nose and mouth; and how well they furnish us with those powers of speech which constitute the most distinctive physical attributes of man.

*Albany; April* 1823.

EXPLANATION OF THE PLATE.

A. Cerebrum.
B. Cerebellum.
C. C. C. Spinal Marrow, with the nerves rising from it.
D. Tongue.
E. Larynx.
F. Lungs.
G. Heart.
H. Stomach.
I. Diaphragm.

1. 1. 1. Par Vagum, arising by a single set of roots, and passing to the larynx, the lungs, heart, and stomach.
2. Superior Laryngeal Branches of the Par Vagum.
3. Recurrent, or Inferior Laryngeal of the Par Vagum.
4. Pulmonic Plexus of the Par Vagum.
5. Cardiac Plexus of the Par Vagum.
6. Gastric Plexus, or Corda Ventriculi of the Par Vagum.
7. Respiratory Nerve, or Portio Dura to the muscles of the face.
8. Branches of the Glosso-Pharyngeal.
9. Lingualis, sending branches to the tongue and to the muscles on the fore-part of the larynx.
10. Origins of the Superior External Respiratory, or Spinal Accessory.
11. Branches of the last nerve to the muscles of the shoulder.
12. 12. 12. Internal Respiratory, or Phrenic to the diaphragm. The origins of this nerve may be seen to pass much higher up than they are generally described.
13. Inferior External Respiratory, to the muscles on the side of the chest.

## Figures and Tables

**PLATE II. f1:**